# On the Condition of the Urine in Typhus and Typhoid Fevers

**Published:** 1853-12

**Authors:** 


The two following articles have each a bearing upon the now vexed question of the identity of fevers. The observations of Dr. Edwards on the urine in continued fevers, is confirmatory of their distinct nature and origin.

In the succeeding article, will be found some views similar to those of Dr. Fenner in relation to the identity of Yellow, Remittent, and Intermittent Fevers. The treatment by large doses of quinine also receives a favorable mention. S. B. H.

*On the Condition of the Urine in Typhus and Typhoid Fevers.*
By George W. Edwards, M. B., Cantab.

Since the researches of observers, and more especially of Dr. Jenner, have established the fact that typhus and typhoid fevers are essentially different diseases, and that the poison of the one does not produce the other, every diagnostic feature becomes of interest. My object in the present paper is to show that a difference in the qualities of the urine exists, and that in the one fever one condition of this secretion, and in the other another condition, supervenes at about the same stage of the disease.

1. *Of the Urine of Typhus Fever.*

The characters of the urine of typhus fever which I desire to point out are—

(1.) *That it is generally pale;*
(2.) *That it is of rather low specific gravity;*
(3.) *That it contains albumen* at an early period of the disease.

The color, an appearance of the urine to the naked eye is indicated in the original tables, and requires no further notice.

It has been remarked by Lehmann that albumen not unfrequently appears for a short time in the urine, in many acute diseases unconnected with affections of the kidneys. It has been found in pneumonia, erysipelas, scarlatina, phthisis, and various other disorders including *typhoid* fevers. "In febrile diseases, properly so called, and particularly among the exanthemata," according to Dr. Begbie,* "a temporary albuminuria may frequently be observed at two distinct and different periods of the disease, the first at nearly the outset of the febrile excitement, and the second toward the termination of the case, when the crisis is passed, and the process of convalescence commenced." This remark does not strictly apply to typhoid fever, as will presently be shown; for when albumen appears in the urine during the progress of that disease, it is only "after the crisis is passed." Of this fact I am assured by my own observations, as well as by the testimony of others.

In *typhus*, however, the urine becomes albuminous at an *early* period of the fever, and I believe that it invariably becomes so in this latter disorder; for out of twenty cases in which I examined the urine, albumen, accompanied with low specific gravity, was present in fourteen, in which the urine was

* Medico-Chirurgical Review, July, 1853.

tested between the sixth and eighteenth day of the fever. In the remaining six, the urine was not tested before the twentieth day, at which period the albuminuria had ceased in all the cases in which I had previously found it to exist; and in five out of these six cases, the specific gravity was below 1014, and therefore below the standard of healthy urine, and as albuminuria is almost constantly attended with low specific gravity, it appears not improbable that in these cases albumen might have been present in the urine at an earlier period than that at which it was tested.

The specific gravity of the urine was low in all the twenty cases which I have recorded, and it was observed, that in the fourteen cases in which albumen was detected, the specific gravity rose gradually, in proportion as the albumen disappeared, until at length it acquired a healthy standard.

The explanation which may be given of this state of the urine is, perhaps, somewhat doubtful, but it appears highly probable that the kidneys become congested at an early period of the fever, and that the effusion of serum is a consequence of the congestion. The results of the post-mortem examinations recorded by Dr. Kirkes, in his position as Medical Registrar at St. Bartholomew's Hospital, at all events favor this view of the matter; for, in six out of seven cases of typhus* which he has reported, the kidneys were found to be in various stages of congestion. In five it is stated that there was "no distinct deposit," and two were "dark, mottled, smooth, friable."

It must not, however, be forgotten, that in typhus the blood is undoubtedly in an altered condition, and that this state of the blood may be the cause of the congestion, or even the direct cause of the effusion of serum.

### 2. *On the Urine in Typhoid Fever.*

Several writers have noticed the more obvious qualities of the urine of *typhoid fever*, namely, its *bright amber color*, and its *high specific gravity*. It has been more recently observed that it deposits on the addition of an acid a *non-albuminous* precipitate at nearly the same period of the disease at which. in cases of *typhus* fever, albumen is present.

On the addition of a drop of *any* mineral, or of acetic acid to a moderate quantity of the urine of a typhoid fever patient in a test tube, a precipitate falls, generally very abundant, like a dense cloud, and to the eye, very like albumen; but upon the addition of an excess of acid, or of a solution of potash, or upon the application of heat, the precipitate entirely disappears; in the last case, however, generally reappearing when the solution becomes cool.

It is almost certain that this precipitate consists of urates, not only from the reactions already mentioned, but because, as Dr. Bence Jones and Dr. Garrod have observed, uric acid crystallizes out of it after some hours. A fact confirmatory of this view is, that in rheumatic fever, where the urates are deposited spontaneously, if the deposit be dissolved in liquor potassæ, it may be again precipitated in a form precisely similar to that produced in the clear high colored urine of typhoid fever, by a drop of acid.

This precipitate is not *peculiar to typhoid fever*, for Dr. Jenner, in one of whose cases at the Hospital for Sick Children, I first observed it, tells me that he has found it in a case of meningitis, and I have myself seen it in the urine of scarlatina at the period when "febrile excitement" prevails. And

---

* *i. e.* Fever without any lesion of the small intestines.

it is well known that salts of uric acid are deposited from the urine in very many febrile conditions of the system.

The precipitate which I have described, takes place in the urine of typhus fever in a very large proportion of cases, and, as I believe, *invariably*. I found it in thirteen out of eighteen cases in which I examined the urine of patients suffering from this disease. The remaining five were all in the end of the third, or in the fourth week, when the disease may be said to have ceased.

In all the cases, albumen was absent, and I believe it will always be found absent, when the kidneys are previously healthy, during the continuance of the fever, or at least during the early period at which albumen appears in typhus; and in the instances in which albumen has been observed in the urine of *typhoid* fever, the crisis of the disease had probably passed.

In those of my cases in which the non-albuminous precipitate was produced, the urine had a high specific gravity, a condition in which it accords with the urine of most diseases attended with febrile excitement, and one in which it again differs from typhus fever.

It is an interesting fact that in cases of typhoid fever fatal before the fourteenth day, the kidneys are not generally congested, as they are in typhus at the same period of the disease; for of thirty four cases recorded at St. Bartholomew's Hospital, by Dr. Kirkes, they are reported as congested only in ten, in all which the disease proved fatal after the fourteenth day. The fact that the kidneys are thus congested at a late stage, agrees with the observations above referred to, that "after the crisis is passed," albumen does occasionally appear in the urine of typhoid fever.

The foregoing facts seem, then, to render the following conclusions highly probable:

(1.) That *albumen* appears in the urine of *every patient* affected with *typhus fever*, at an *early* period of the disease, and that in such cases the urine is of low specific gravity.

(2.) That in the *early stages of typhoid fever*, albumen is absent, while, on the other hand, the urine has an *unnaturally high specific gravity*, with excess of one of its natural constituents, viz., urate of ammonia; and

(3.) That the state of the kidneys in either disease corresponds with the condition of the urine, these organs being *congested* in *typhus*, and generally *healthy* in *typhoid* fever.—*Monthly Journal of Med. Science*, from *N. Y. Jour. of Medicine*.

